# Biophysical markers of the peripheral vasoconstriction response to pain in sickle cell disease

**DOI:** 10.1371/journal.pone.0178353

**Published:** 2017-05-24

**Authors:** Patjanaporn Chalacheva, Maha Khaleel, John Sunwoo, Payal Shah, Jon A. Detterich, Roberta M. Kato, Wanwara Thuptimdang, Herbert J. Meiselman, Richard Sposto, Jennie Tsao, John C. Wood, Lonnie Zeltzer, Thomas D. Coates, Michael C. K. Khoo

**Affiliations:** 1 Department of Biomedical Engineering, University of Southern California, Los Angeles, California, United States of America; 2 Division of Hematology, Oncology and Blood & Marrow Transplantation, Children's Center for Cancer and Blood Diseases, Children's Hospital Los Angeles, Los Angeles, California, United States of America; 3 Division of Cardiology, Children’s Hospital Los Angeles, Los Angeles, California, United States of America; 4 Department of Pediatrics, Keck School of Medicine, University of Southern California, Los Angeles, California, United States of America; 5 Division of Pulmonology, Children’s Hospital Los Angeles, Los Angeles, California, United States of America; 6 Department of Physiology and Biophysics, Keck School of Medicine, University of Southern California, Los Angeles, California, United States of America; 7 Department of Preventative Medicine, Keck School of Medicine, University of Southern California, Los Angeles, California, United States of America; 8 Pediatric Pain Program, David Geffen School of Medicine, University of California at Los Angeles, California, United States of America; Université Claude Bernard Lyon 1, FRANCE

## Abstract

Painful vaso-occlusive crisis (VOC), a complication of sickle cell disease (SCD), occurs when sickled red blood cells obstruct flow in the microvasculature. We postulated that exaggerated sympathetically mediated vasoconstriction, endothelial dysfunction and the synergistic interaction between these two factors act together to reduce microvascular flow, promoting regional vaso-occlusions, setting the stage for VOC. We previously found that SCD subjects had stronger vasoconstriction response to pulses of heat-induced pain compared to controls but the relative degrees to which autonomic dysregulation, peripheral vascular dysfunction and their interaction are present in SCD remain unknown. In the present study, we employed a mathematical model to decompose the total vasoconstriction response to pain into: 1) the neurogenic component, 2) the vascular response to blood pressure, 3) respiratory coupling and 4) neurogenic-vascular interaction. The model allowed us to quantify the contribution of each component to the total vasoconstriction response. The most salient features of the components were extracted to represent biophysical markers of autonomic and vascular impairment in SCD and controls. These markers provide a means of phenotyping severity of disease in sickle-cell anemia that is based more on underlying physiology than on genotype. The marker of the vascular component (BM_v_) showed stronger contribution to vasoconstriction in SCD than controls (p = 0.0409), suggesting a dominant myogenic response in the SCD subjects as a consequence of endothelial dysfunction. The marker of neurogenic-vascular interaction (BM_n-v_) revealed that the interaction reinforced vasoconstriction in SCD but produced vasodilatory response in controls (p = 0.0167). This marked difference in BM_n-v_ suggests that it is the most sensitive marker for quantifying combined alterations in autonomic and vascular function in SCD in response to heat-induced pain.

## Introduction

Sickle cell disease (SCD) is an inherited blood disease, characterized by episodes of excruciating pain, progressive end organ dysfunction, and premature death. Upon releasing oxygen, sickle hemoglobin (HbS) polymerizes, transforming flexible biconcave disc shaped red blood cells into rigid sickle shaped cells [1]. These sickled red blood cells are rigid and sticky; they can obstruct blood flow in tiny capillaries or adhere to the vessel wall, narrowing the blood vessel, which may result in microvascular occlusion. Extensive microvascular occlusion eventually leads to vaso-occlusive crisis (VOC), often resulting in episodes of pain, organ damage or death. To date, the exact chain of events and physiological mechanisms that lead to the transformation from steady state to VOC remain unclear. Eaton and Hofrichter [2] showed that there is a time delay between oxygen release and the polymerization of HbS and hypothesized that any factors that prolong the time it takes for these red blood cells to traverse the microvasculature before HbS polymerization causes the red cells to become rigid can increase the likelihood of vaso-occlusion.

Abnormal sympathetic control of peripheral vascular resistance is likely one of the primary factors that can reduce peripheral blood flow in response to certain autonomic stimuli. Our group previously showed that SCD had higher probability of vasoconstriction following a spontaneous sigh compared to healthy subjects, suggesting that SCD subjects have exaggerated sympathetic responses to respiration [3]. Pain is known to activate the sympathetic nervous system, as demonstrated by studies measuring muscle sympathetic nerve activity [4–6], and the increase in sympathetic drive can lead to peripheral vasoconstriction. We previously found that pain, induced through the delivery of dynamic pulses of heat to the skin, led to stronger phasic vasoconstriction responses in SCD compared to non-SCD subjects [7]. So while pain is thought to be a consequence of VOC, the pain itself may trigger a cascade of events that leads to full-scale VOC by promoting regional vasoconstriction.

It is also well-established that SCD subjects have impaired endothelial function [8] due in part to deficient bioavailability of nitric oxide, an endogenous vasodilator [9]. Detterich et al. [10] showed that SCD had marked decrease in flow-mediated dilation, indicating lower capacity to vasodilate in response to an increase in flow. Acute pain can contribute indirectly to reduced microvascular blood flow in the following manner. Sympathetic activity triggered by pain increases heart rate and stroke volume–which, in conjunction with peripheral vasoconstriction, increase blood pressure. Under ideal circumstances, this blood pressure increase would produce peripheral vasodilation through the sympathetically-mediated baroreflex control of vascular resistance, as well as through flow-mediated dilation. However, in subjects who have impaired endothelial function and blunted baroreflex function, the pain-mediated blood pressure increase could instead produce further peripheral vasoconstriction. Synergistic interaction between impaired local vascular function and the exaggerated sympathetically-mediated vasoconstrictor response could further reduce peripheral blood flow, setting the stage for VOC.

In this study, we applied a “systems” approach, in which we related the vasoconstriction responses (considered the “output”) to the key “inputs” that most strongly influenced these responses during application of heat-induced pain pulses. By so doing, we were able to derive the relative contributions of autonomic dysregulation, peripheral vascular dysregulation and their interaction present in SCD and controls. More specifically, this approach was implemented using a mathematical model to quantify the contribution of each input to the peripheral vasoconstriction response to pain. From the estimated model parameters, we extracted compact descriptors (“markers”) that quantify the most salient features of the vasoconstriction responses triggered by pain. These model-based markers provided a novel basis for quantifying the impairments in autonomic and vascular control in SCD subjects.

## Methods

### Participants

All experiments were conducted at Children’s Hospital Los Angeles (CHLA). The study protocol was approved by the Committee on Clinical Investigations (institutional review board of CHLA). African American participants, with age range between 13 and 55 years old, were selected from healthy (AA) subjects, sickle cell trait (heterozygous hemoglobin AS) subjects and SCD subjects (homozygous SS and S-β^0^ thalassemia). Subjects were excluded if they had pain crisis or hospitalization in the past two weeks; had diabetes, ischemic heart disease and/or acute or chronic illness that may compromise subject safety or data integrity; were on short acting opioids; had neurological disorders affecting sensation; had skin abnormality/abrasion over sites of stimulus; or were unable to follow instructions. Written informed consent/assent was obtained before participation of the study. We obtained IRB approved assent for the minors and IRB approved consent from the parents if the subject was younger than 14 years old in accordance with protocols established by the CHLA IRB. Subjects were classified into two groups: non-SCD (healthy and sickle cell trait subjects) and SCD. Data acquired from total of 45 subjects were studied. Subject characteristics are summarized in Table 1.

**Table 1:** Subject characteristics and baseline physiological measurements.

	Non-SCD (N = 23)	SCD (N = 22)	P-value^†^
Genotype	AA = 10, AS = 13	SS = 21, Sβ^0^ = 1	-
Age (years)	30.5 ± 2.5	20.5 ± 1.3	0.0010 *
Male/Female	9 / 14	8 / 14	0.8482 ^‡^
Hgb (g/dL)	13.4 ± 0.3	10.1 ± 0.3	< 0.0001 *
HbS (%)^#^	-	37.0 ± 6.9	-
Reticulocyte count (%)	1.4 ± 0.1	8.0 ± 1.1	< 0.0001 *
Pain threshold (°C)	44.2 ± 0.7	43.0 ± 2.8	0.2153
Pain tolerance (°C)	48.4 ± 0.4	48.1 ± 0.3	0.4954
HR (bpm)	69.2 ± 2.0	83.2 ± 2.4	< 0.0001 *
SBP (mmHg)	127.2 ± 3.6	112.6 ± 3.4	0.0051 *
DBP (mmHg)	75.3 ± 1.8	67.0 ± 1.1	0.0004 *
SpO_2_ (%)	95.4 ± 0.4	95.1 ± 0.4	0.4750
FBV (%)	66.9 ± 5.6	56.1 ± 4.4	0.1362

*Definition of abbreviations*: Hgb = hemoglobin concentration; HbS = hemoglobin S; HR = heart rate; SBP = systolic blood pressure; DBP = diastolic blood pressure; SpO_2_ = oxygen saturation by pulse oximetry; FBV = finger blood volume derived from photoplethysmogram. Data show mean ± SEM.

* p < 0.05.

^†^ P-value from Student’s t-test

^‡^ p-value from Chi-squared test

^#^ HbS that can be sickled

### Protocols

Measurements were carried out in the autonomic laboratory (a quiet, dimmed light, temperature-controlled room) where the participant rested comfortably at 45-degree angle on a cushioned chair with arm and leg supports. After at least 5 minutes of quiet and stable baseline recording was obtained, the subject received 6 heat-induced pain pulses, separated by 30 seconds, from the computer controlled thermode (TSA-II NeuroSensory Analyzer, Medoc Advanced Medical Systems, Inc., USA) on the right forearm. For each of the first 3 heat pulses, the pain pulse was delivered with increasing intensity (increasing temperature) at a fixed rate of 1°C/sec until the subject indicated that the heat was perceived as painful (pain threshold) then the thermode temperature immediately returned to its resting temperature of 32°C. Similarly for each of the last 3 pulses, we let the temperature increase but this time we stopped increasing the temperature when the subject indicated that pain was perceived as intolerable (pain tolerance). For safety, the temperature could not increase beyond 52°C. This preliminary approach established the “pain threshold” and “pain tolerance” temperature for each individual subject. These individualized temperatures ensured that the subjects felt the pain that was calibrated to their own perception. The average pain threshold and pain tolerance temperatures are reported in Table 1.

Other physiological measurements included respiration (thorax and abdominal bands, zRip DuraBelt, Philips), electrocardiogram (ECG), continuous blood pressure (Nexfin; BMEYE, Amsterdam, The Netherlands), photoplethysmogram (Nonin Medical Inc., USA) and microvascular flow using laser Doppler flowmeter (PeriFlux System, Perimed, Sweden). Blood pressure and photoplethysmogram (PPG) were measured on the middle finger and the thumb on the contralateral hand, respectively. The laser Doppler sensors were placed on both index fingers 3 mm proximal to the nail bed. All measurements were acquired synchronously and continuously through Biopac MP150 data acquisition system (Biopac, USA) at 250 Hz.

### Data preprocessing

Tidal volume (V_T_) was derived from both thorax and abdominal bands as described in Sackner et al. [11]. The beat-to-beat variables were extracted using the R-waves on the ECG. R-R interval (RRI) was defined as the time between two consecutive R-waves on the ECG. Diastolic and systolic blood pressure (DBP and SBP) were the nadir and the peak of the blood pressure pulse within each RRI. Beat-averaged arterial blood pressure (MAP) was the average of the entire blood pressure pulse from one DBP to the immediately following DBP. The pulse amplitude of the PPG was the difference between the peak and nadir of PPG pulse within each RRI. PPG pulse amplitude (PPGamp) reflects pulsatile change in finger blood volume (ΔFBV) caused by arterial blood flow around the fingertip [12,13]. Thus, decreases/increases in PPGamp represent vasoconstriction/dilation. Since PPGamp is a relative measurement (in arbitrary units), ΔFBV was defined as PPGamp normalized to its own 95^th^ percentile value, and expressed as a percent.

### Functional mechanisms of pain-induced vasoconstriction

**Fig 1:**
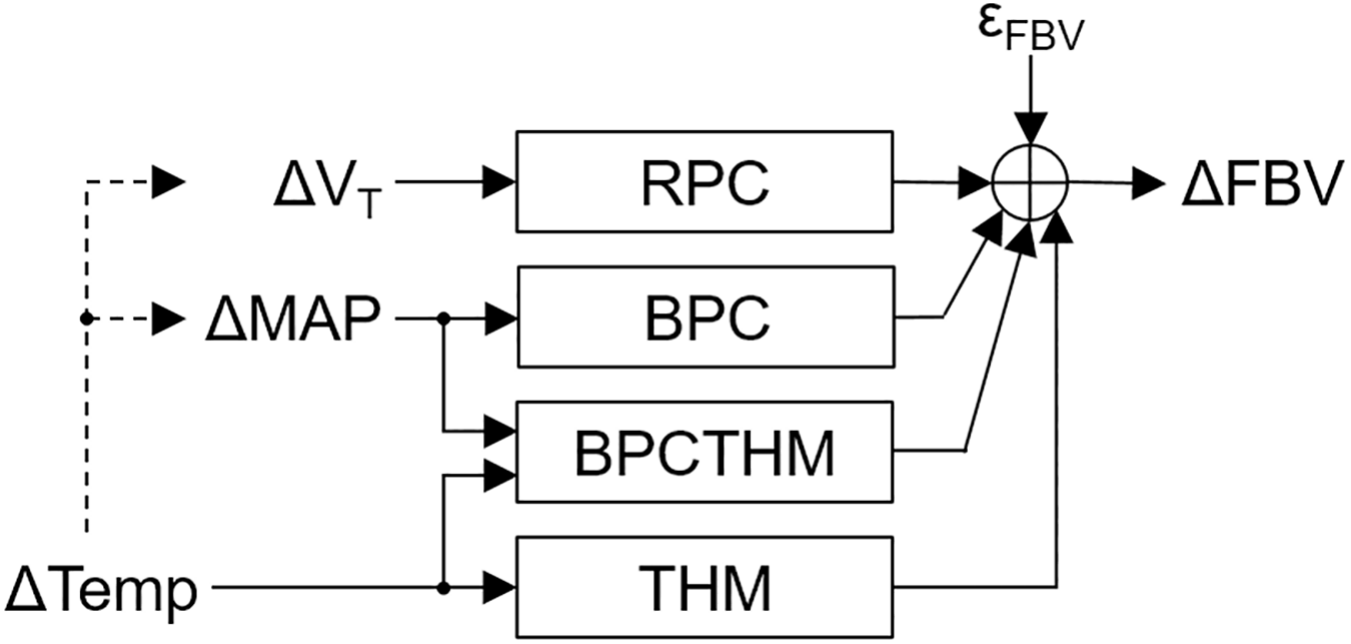
Schematic diagram of the model of peripheral vasoconstriction response to heat-induced pain. Functional relationships between changes in temperature (ΔTemp), blood pressure (ΔMAP) and respiration (ΔV_T_) as inputs and changes in finger blood volume (ΔFBV) as the output. ε_FBV_ denotes extraneous fluctuations on FBV that are not captured by the model.

We employed a mathematical modeling approach to unravel the relationships between the physiological response of interest and the experimentally applied stimulus in the face of other spontaneous stimuli. This approach has been used in other applications. For more details the readers are referred to [14,15]. The mathematical model allows us to computationally partition the overall physiological system regulating the vasoconstriction response into specific stimulus-response relations in order to understand their individual contributions and the interactions between these stimuli. In this study, we determined how heat-induced pain (external stimuli) affected peripheral vasoconstriction. Based on our understanding of the physiology regulating FBV, the proposed model (Fig 1) consisted of 4 components: 1) blood pressure coupling (BPC): blood pressure affects ΔFBV through sympathetically-mediated baroreflex control of peripheral resistance [16] as well as through local regulation of blood flow [17,18]; 2) respiratory coupling (RPC): respiration is known to modulate sympathetic neural activity [19,20] and changes in breathing induced by pain, e.g. sighs and breath holds, may elicit changes in peripheral resistance [3,21]; 3) neurogenic thermal pain coupling (THM): heat-induced pain affected ΔFBV through sympathetic modulation directly as well as through its modulation on MAP and V_T_; and 4) the neurogenic-vascular interaction (BPCTHM): the interaction effect between ΔMAP and ΔTemp on ΔFBV. The mathematical representation of the model incorporating all the above factors is displayed below:

$$\begin{array}{l} \Delta FBV(t)=\sum_{i=0}^{M-1}h_{RPC}(i)\Delta V_{T}(t-i-T_{RPC})\\ \;+\sum_{i=0}^{M-1}h_{BPC}(i)\Delta MAP(t-i-T_{BPC})\\ \;+\sum_{i=0}^{M_{THM}-1}h_{THM}(i)\Delta Temp(t-i-T_{THM})\\ \;+\sum_{i=0}^{M-1}\sum_{j=0}^{M_{THM}-1}h_{BPCTHM}(i,j)\Delta MAP(t-i-T_{BPC})\Delta Temp(t-j-T_{THM})\\ \;+\varepsilon_{FBV}(t). \end{array}$$ (1)

*h*_*BPC*_, *h*_*RPC*_, *h*_*THM*_ and *h*_*BPCTHM*_ represent “standardized” FBV responses to a unit pulse increases in the corresponding inputs. The last term in Eq (1) represents the “residuals”–the contributions to ΔFBV that cannot be accounted for by the model. The model characterized by Eq (1) was selected after a systematic process of comparing its goodness of fit with all datasets, relative to other candidate models that incorporated only a subset of the inputs. Details on the model selection and estimation procedures are given in S1 File.

### Dynamic interaction between pain and blood pressure

Unlike interaction in a statistical model, dynamic interaction between two signals is dependent on the time at which the changes in two signals occur relative to each other. For example, the dynamics of the interaction effect due to changes in Temp and MAP that are 1 second apart can be different from that when Temp and MAP are 2 seconds apart. To demonstrate how Temp interacted with MAP that then affected FBV, we simulated Temp and MAP pulses. We let the Temp pulse be 10-second long with a pulse height of 20°C, which was approximately the average height of the experimental heat pulses. We also let the MAP pulse be 10 seconds in duration. Since the timing between the Temp and MAP pulses was crucial, we determined the time lag between Temp and MAP where the two signals were most positively correlated using cross-correlation, which we found to be 2 seconds. We also determined the simulated MAP pulse height from the data by averaging the changes in MAP magnitude as a result of heat pulse, which we found to be 10 mmHg.

Next, we convolved the simulated Temp and MAP pulses with the standardized interaction component, *h*_*BPCTHM*_, of each subject to generate the interaction effect between Temp and MAP on FBV using Eq (2).

$$\Delta FBV_{BPCTHM}(t)=\sum_{i=0}^{M-1}\sum_{j=0}^{M_{THM}-1}h_{BPCTHM}(i,j)\Delta MAP(t-i-T_{BPC})\Delta Temp(t-j-T_{THM})$$ (2)

### Biophysical markers

We extracted compact descriptors from the model to quantify the dynamics of the standardized FBV response components and used them as the biophysical markers to differentiate the FBV response to heat-induced pain between SCD and non-SCD.

1. BM_n_: The area of *h*_*THM*_ from 0 to 15 seconds was selected to represent the initial neurogenic vasoconstriction caused by the direct effect of pain.
2. BM_v_: The area of *h*_*BPC*_ from 1 to 5 seconds was selected to represent the vascular response to an increase in blood pressure.
3. BM_n-v_: The total area of the interaction response to the simulated Temp and MAP pulses was used to quantify the FBV response to the neurogenic-vascular interaction.

For all 3 descriptors, negative values signify vasoconstriction while positive values signify vasodilation; and the more negative/positive the values, the stronger the vasoconstriction/vasodilation. For ease of further reading, abbreviations that will be frequently used are listed in Table 2.

**Table 2:** List of abbreviations.

SCD	Sickle cell disease
Temp	Temperature
V_T_	Tidal volume
MAP	Mean arterial pressure
FBV	Finger blood volume
BPC	Blood pressure coupling
RPC	Respiratory coupling
THM	Neurogenic thermal pain coupling
BPCTHM	Neurogenic-vascular interaction
BM_n_	Biophysical marker representing the neurogenic vasoconstriction caused by the direct effect of pain
BM_v_	Biophysical marker representing the vascular response to an increase in blood pressure
BM_n-v_	Biophysical marker representing the response to the neurogenic-vascular interaction

### Statistical tests

All results were reported in mean ± standard error of the mean (SEM). Student’s t-test was used to test for group difference in the subject characteristics and baseline physiological parameters. Multiple linear regression was applied to the model-derived biophysical markers to test for group difference after adjusting for age and sex. All statistical analyses were performed using JMP statistical software, version 12.1 (SAS Institute Inc., Cary, NC).

## Results

### Subject characteristics and baseline parameters

Table 1 summarizes subject characteristics and baseline physiological measurements. SCD were generally younger than non-SCD subjects. There were more female subjects overall but the male/female ratio was similar in both groups. SCD had lower hemoglobin concentration and higher reticulocyte count than non-SCD subjects. Baseline physiological measurements showed that SCD had higher heart rate but lower blood pressure than non-SCD. Baseline arterial oxygen saturation and FBV were similar in both groups.

### Sample time-series

**Fig 2:**
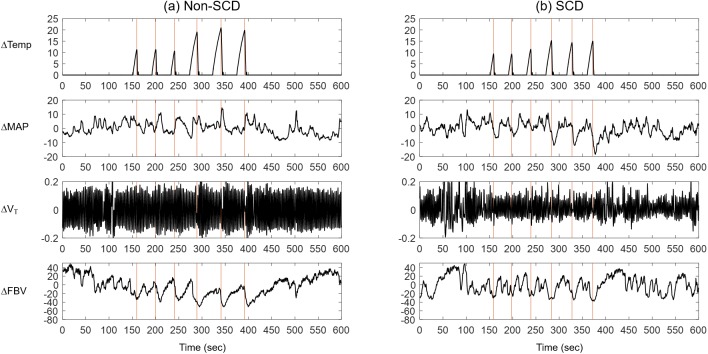
**Representative data recorded during a test procedure in (a) a non-SCD subject and (b) a SCD subject.** The top row shows changes in temperature (ΔTemp, °C). 0°C indicates no heat was delivered. Row 2 shows changes in beat-averaged blood pressure (MAP, mmHg). Row 3 shows changes in tidal volume (ΔV_T_, L). The bottom row shows corresponding changes in finger blood volume (ΔFBV, %).

Representative data segments obtained from a non-SCD and a SCD subjects are shown in Fig 2A and 2B, respectively. The top row shows heat-induced pain delivered during the test procedure. ΔTemp = 0°C indicates that no pain was delivered. MAP tended to exhibit fluctuations following each heat pulse. The V_T_ traces show brief breath holding at the end of each pain pulse. This reaction was common among many subjects. Other reactions to pain included taking deep breaths and hypoventilation during the pain task. Nonetheless, the most remarkable changes in physiological measurement induced by heat pain were the changes in FBV (bottom row). It is evident, in this example, that each pain pulse corresponded to a drop in FBV, suggesting vasoconstriction following each pain pulse, and the height of the pain pulse was correlated with the degree of vasoconstriction. A similar response was observed in the microvascular flow measured by the laser Doppler flowmeter (S1 Fig), confirming that negative changes in FBV represented reductions in microvascular flow resulting from vasoconstriction.

**Fig 3:**
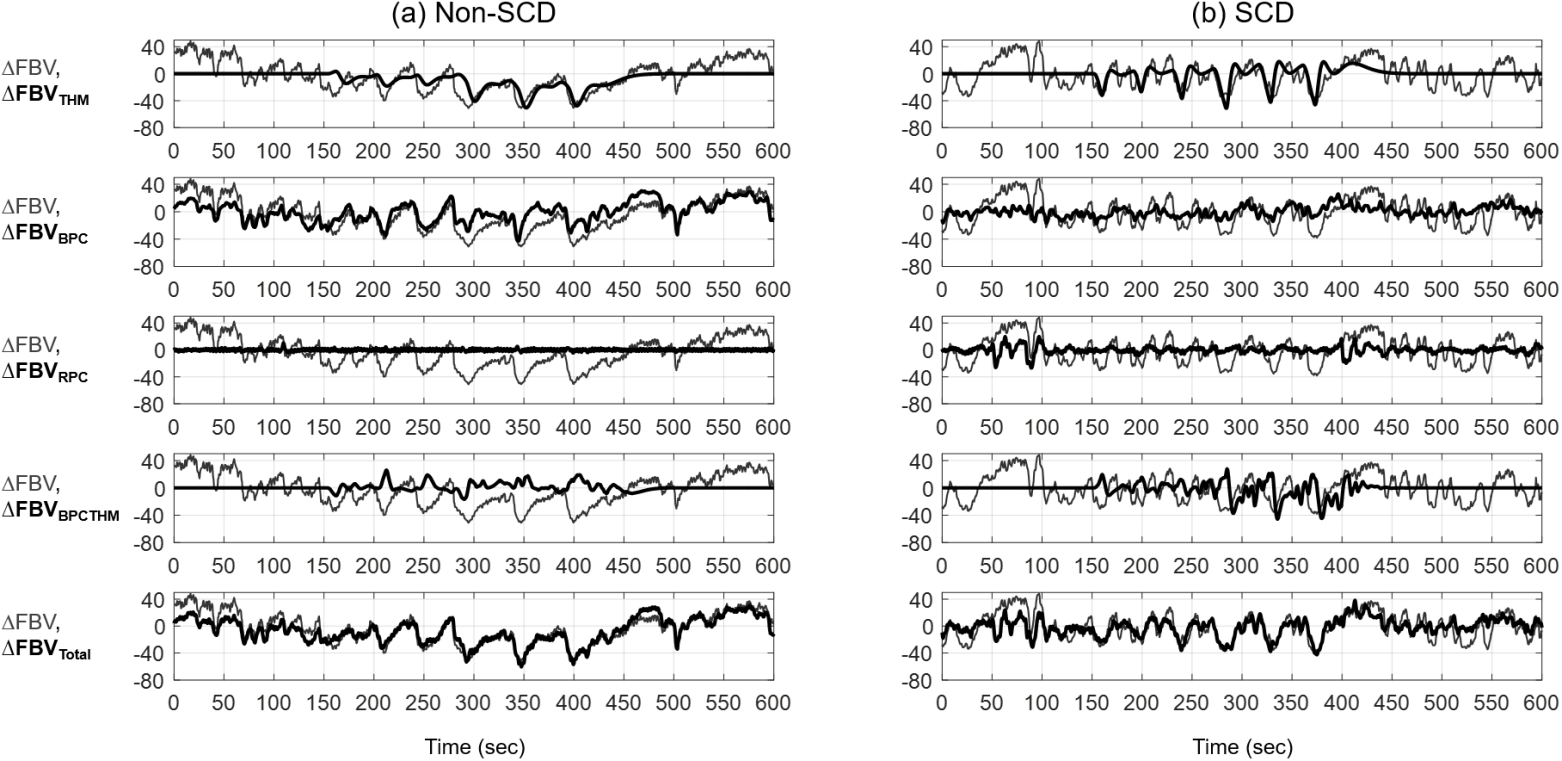
FBV response partitioned into contributions from each model component. Predicted ΔFBV by each model component (thick lines) plotted on top of the measured ΔFBV (thin lines) during a test procedure in (a) a non-SCD subject and (b) a SCD subject, showing the relative contribution from each component on the total ΔFBV. The bottom row shows the sum of the predicted ΔFBV by all model components (thick line).

Fig 3 shows predicted FBV response by each model component (thick lines) plotted on top of the measured FBV response (thin lines) of (a) a non-SCD subject and (b) a SCD subject. It is clear from the figure that THM and BPC were the main contributing components to the total vasoconstriction. The contribution of respiration on vasoconstriction was small relative to other model components so we did not derive any biophysical markers from the RPC component. However, this component was kept in the model to account for any respiratory effect, especially the effect of irregular breathing elicited by pain on FBV. The BPCTHM component contributed moderately to the changes in FBV. The total predicted response (bottom row) is the sum of each individual prediction. The total predicted response fit the measured response well, indicating that the mathematical model was able to capture the fluctuations in FBV.

### Model-based markers of pain-induced vasoconstriction

**Fig 4:**
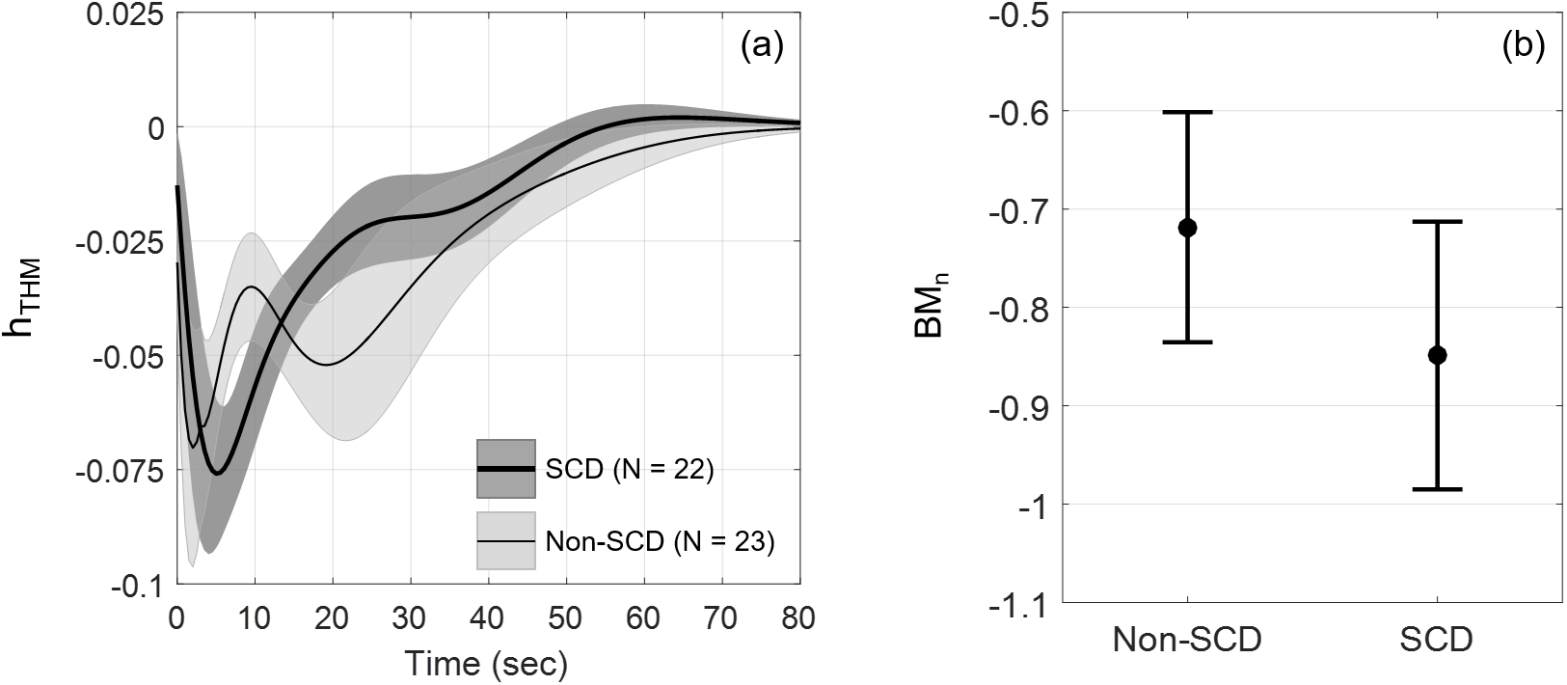
Standardized FBV responses to a pain pulse and derived biophysical marker BM_n_. (a) *h*_*THM*_, standardized FBV responses to a pain pulse (mean ± SEM) in SCD (N = 22, thick line) and non-SCD (N = 23, thin line); (b) biophysical marker BM_n_ capturing the initial neurogenic vasoconstriction caused by the direct effect of pain. Error bars show mean ± SEM. SCD had slightly stronger vasoconstriction than non-SCD (not statistically significant).

The group-averaged standardized responses to the direct effect of heat-induced pain on FBV, *h*_*THM*_, are shown in Fig 4A. Both groups showed negative response, i.e. vasoconstriction, to pain after accounting for the effect of blood pressure, respiration and interaction between ΔTemp and ΔMAP. Non-SCD subjects exhibited biphasic response, a quick return from the maximal vasoconstriction followed by more prolonged but smaller vasoconstriction, while SCD subjects showed a more gradual return from the maximal vasoconstriction. The derived biophysical marker BM_n_ captures the initial neurogenic vasoconstriction caused by the direct effect of pain (Fig 4B). SCD showed slightly stronger vasoconstriction but was not statistically different from non-SCD.

**Fig 5:**
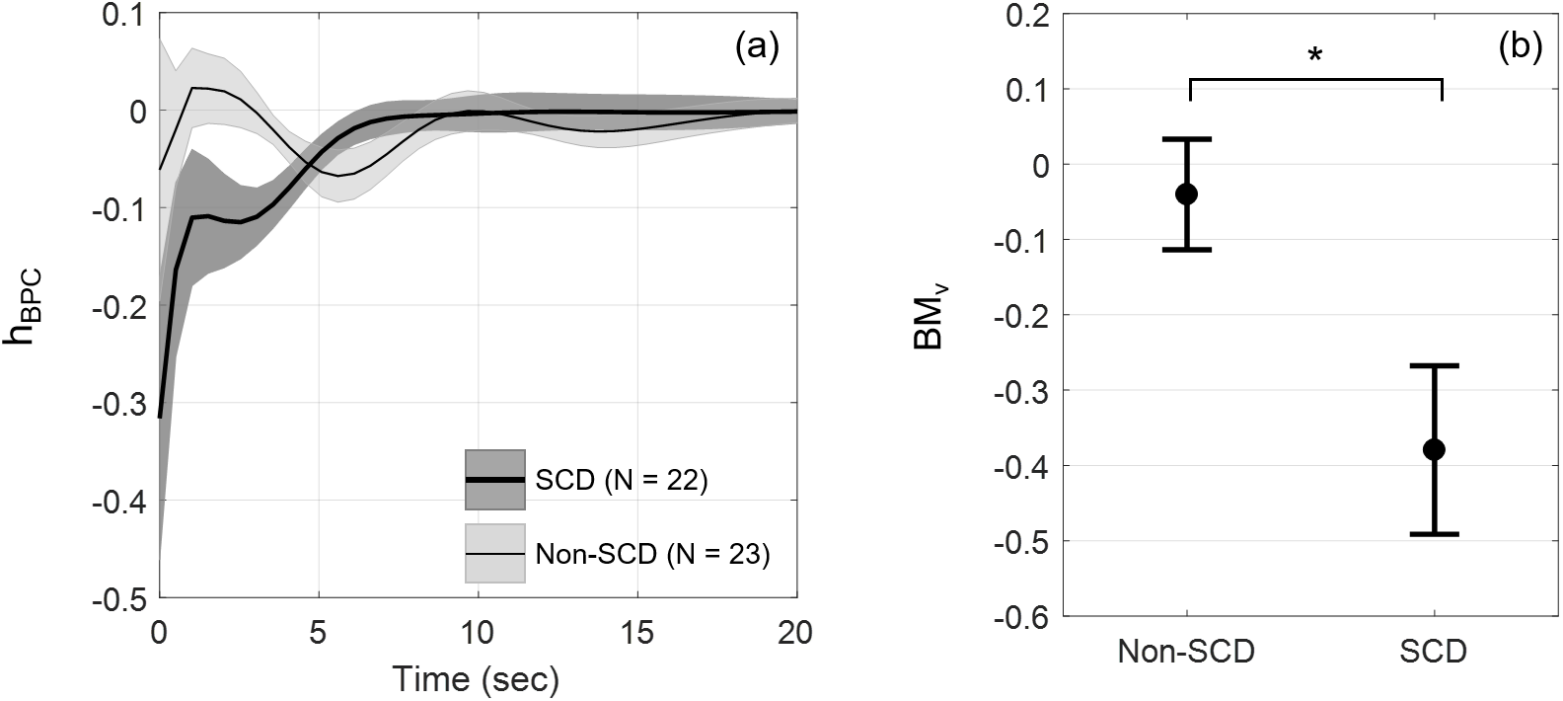
Standardized FBV responses to a blood pressure pulse and derived biophysical marker BM_v_. (a) *h*_*BPC*_, standardized FBV responses to a blood pressure increase (mean ± SEM) of SCD (N = 22, thick line) and non-SCD (N = 23, thin line); (b) biophysical marker BM_v_ reflecting vasoconstriction as a result of blood pressure increase. Error bars show mean ± SEM. SCD had significantly stronger vasoconstriction than non-SCD after adjusting for age and sex (p = 0.0409).

Another major contributing mechanism to vasoconstriction was BPC, which relates ΔMAP to ΔFBV. The group-averaged standardized responses of BPC to a blood pressure pulse, *h*_*BPC*_, are shown in Fig 5A. There was a clear distinction between the BPC dynamics of SCD and non-SCD subjects. SCD showed negative response, i.e. vasoconstriction, which then returned to zero after 6 seconds. On the contrary, the non-SCD response showed oscillations around zero. The estimated BPC likely represents the myogenic response to pressure changes rather than the baroreflex control of peripheral resistance, because the latter should counteract the increase in blood pressure by vasodilation. Fig 5B shows the biophysical marker BM_v_, representing the vascular response to blood pressure increase. Multiple linear regression produced F(3,41) = 3.204, p = 0.0330 and revealed that SCD had significantly stronger vasoconstriction due to an increase in MAP than non-SCD (p = 0.0409), whose FBV remained relatively unchanged, after adjusting for age and sex. The stronger vasoconstriction induced by blood pressure rise suggests altered peripheral blood flow regulation in SCD.

**Fig 6:**
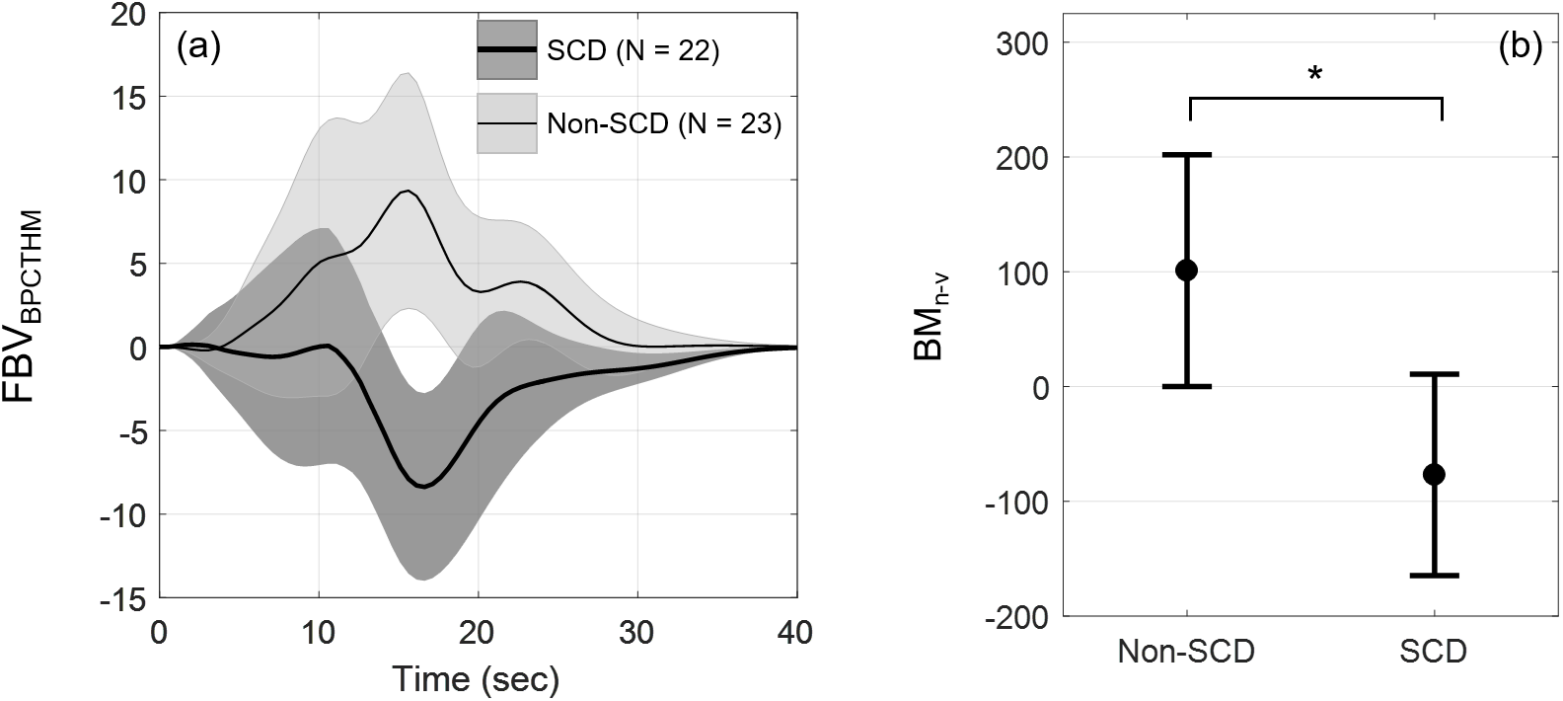
FBV responses of the interaction between pain and blood pressure and derived biophysical marker BM_n-v_. (a) *FBV*_*BPCTHM*_, responses of the interaction between the simulated Temp and MAP pulses (mean ± SEM) in SCD (N = 22, thick line) and non-SCD (N = 23, thin line); (b) biophysical marker BM_n-v_ reflecting total change in FBV as a result of the interaction between the two inputs. Error bars show mean ± SEM. SCD vasoconstricted while non-SCD vasodilated (p = 0.0167).

Fig 6A shows the FBV response to the interaction between the simulated Temp and MAP pulses. The interaction effect revealed distinct responses between the two groups: non-SCD subjects vasodilated as a result of the interaction while SCD subjects vasoconstricted. Hence, the vasodilation due to the interaction effect in non-SCD helped counteract the vasoconstriction induced by the heat pain while reinforced vasoconstriction in SCD group. The biophysical marker BM_n-v_ represents the total change in FBV as a result of the neuro-vascular interaction (Fig 6B). Multiple linear regression produced F(3,41) = 3.138, p = 0.0355. The total change in FBV as a result of the interaction effect was different between subject groups after adjusting for age and sex (p = 0.0167): non-SCD had positive change in FBV (vasodilation) while SCD had negative change in FBV (vasoconstriction). There was also significant age effect–older subjects vasoconstricted as a result of the Temp-MAP interaction and vice versa (p = 0.0147).

## Discussion

We previously reported that SCD subjects had stronger vasoconstriction in response to heat-induced pain than non-SCD controls [7]. Since pain triggers rapid changes in blood pressure and respiration, and these auxiliary changes can also lead to peripheral vasoconstriction, we sought to quantify the relative contributions of these additional inputs to pain-induced vasoconstriction, and thus determine how these contributions were different in SCD vs controls. To accomplish this goal, we used a mathematical model to computationally decompose the peripheral vasoconstriction response into individual component signals, each correlated with its corresponding input time-series. The model provided the rigorous framework that allowed us to extract meaningful information from time-series datasets where both the inputs and output were measured simultaneously. This approach is analogous to multiple regression analysis, except that in this case, the regression coefficients themselves vary with time. We found that: 1) the primary contributors to the vasoconstriction response to heat-induced pain were the neurogenic component (THM), the vascular response to changes in blood pressure (BPC), and the neurogenic-vascular interaction (BPCTHM); 2) the respiratory contribution was considerably smaller than the other components; 3) the vascular response to blood pressure increase contributed further to vasoconstriction in SCD than non-SCD; and 4) the neurogenic-vascular interaction reinforced vasoconstriction in SCD whereas in non-SCD controls, the interaction produced a vasodilatory component that acted to offset the other vasoconstrictive components. To facilitate the comparison of the magnitude and dynamics of these estimated components across subjects, we extracted compact descriptors from the model that constituted markers BM_n_, BM_v_ and BM_n-v_.

### Neurogenic component

In our model, we attributed the direct effect of pain on vasoconstriction to the neurogenic component (THM). The biophysical marker of the sympathetically-mediated neurogenic response, BM_n_, although not statistically significant, showed a tendency to be stronger in SCD as reflected by the larger maximal vasoconstriction (Fig 4). In a separate analysis to assess the autonomic modulations of the heart rate [22], we also found an increase in the ratio of low-frequency power to high-frequency power of RRI fluctuations during pain stimulation compared to baseline. Both findings indicate a tipping of sympathovagal balance towards sympathetic predominance as a result of pain. Other studies also reported abnormal autonomic function in sickle cell diseases, mostly through the assessment of heart rate variability in response to various tests such as cardiovascular autonomic function tests [23], mental challenges [24] and exposure to transient hypoxia [3] while fewer investigated the autonomic regulation of peripheral resistance [25,26]. These studies revealed that SCD have decreased cardiac parasympathetic activity/reactivity but increased sympathetic drive to the heart and peripheries.

### Vascular response to blood pressure increase

The second component that contributed significantly to the vasoconstriction response was the vascular response to changes in blood pressure component (BPC). This response was quantified by the marker BM_v_. Vasomotor function is influenced by vasoactive substances produced by endothelial cells as well as the myogenic response to changes in transmural pressure [27]. Huang et al. [28] showed that in isolated no-flow muscle arteriole of Wistar-Kyoto rats, the diameter of the vessel decreased with increasing pressure; but when the arteriole was denuded of endothelial cells, the decrease in diameter became larger with increasing pressure. These findings suggest that the myogenic response is stronger when endothelial function is impaired and too weak to buffer the opposing effect. In our study, we found that SCD vasoconstricted more strongly in response to an increase in blood pressure than non-SCD, i.e. BM_v_ was substantially more negative in SCD vs non-SCD (Fig 5). This suggests a dominant myogenic response in the SCD subjects, possibly a consequence of endothelial dysfunction. Endothelial dysfunction results in nitric oxide depletion and elevated endothelin-1, thus impairing the ability of arterioles to dilate while promoting vasoconstriction. Previous studies showed evidence of endothelial dysfunction in SCD as their flow-mediated dilation was significantly lower than healthy controls [8,10,29].

### Neurogenic-vascular interaction

The neurogenic-vascular interaction component, quantified by the marker BM_n-v_, may be ascribed to the interrelationship between autonomic nervous system and vascular function as endothelial and autonomic dysfunction often co-exist [30,31]. Previous studies revealed that central and peripheral autonomic control as well as regulation of vascular function share the same regulatory pathways [32,33]. In addition, Sverrisdóttir et al. [34] found negative correlation between muscle sympathetic neural activity and reactive hyperemia index assessed by pulse amplitude tonometry at the fingertip in healthy subjects. Similarly, another study found that sympathetic stimulation by applying lower body negative pressure resulted in attenuated flow-mediated dilation response through an alpha-adrenergic mechanism [35]. While these findings imply sympathetic modulation of vascular function, the reverse–the sympathetic outflow being influenced by changes in endothelial function–is also possible. Animal studies showed that centrally administered endothelin-1 in rats led to activation of central as well as peripheral sympathetic nervous system [36,37] and acute intravenous administration of nitric oxide synthase inhibitor resulted in increased renal sympathetic nerve activity [38].

Our finding that the neurogenic-vascular interaction in SCD enhanced vasoconstriction but acted to offset vasoconstriction in non-SCD (Fig 6) is an important one. In terms of the biophysical marker, BM_n-v_ was predominantly positive in numerical value in the control group, but largely negative (representing vasoconstriction) in the SCD group. This marked difference in values of BM_n-v_ suggests this marker is the most sensitive, and perhaps the most useful from a clinical standpoint, for quantifying combined alterations in autonomic and vascular function in SCD. Future studies are needed to determine the clinical utility of this marker.

### Study limitations

While the mathematical model provides a way to derive biophysical markers from the measured data, there are limitations associated with this approach. First, our model considered the primary physiological mechanisms that contributed to pain-triggered vasoconstriction but there could be missing components that could not be estimated from our data because the associated inputs were not measured in our studies. We also assumed that pain, blood pressure and respiration were linearly related to changes in FBV. Although the model showed reasonably good fit, the assumption of linear relationships between the inputs and the vasoconstriction response is necessarily a simplification of the underlying reality. Lastly, Khaleel et al. [7] reported vasoconstriction due to anticipation prior to receiving pain. The anticipatory effect was in part accounted for in the model by changes in blood pressure, although this could not be directly measured.

Other limitations of the study not directly related to modeling included the differences in subject characteristics between SCD and non-SCD: baseline heart rate, blood pressure and age. Higher heart rate in SCD could be in part ascribed to the effect of anemia [39]. Lower blood pressure in SCD could be ascribed to decreased vascular resistance as a result lower blood viscosity due to anemia as well as new vessel recruitment and formation of collaterals and arteriovenous shunts [39]. At the same time, higher blood pressure in non-SCD group could be influenced by age. The age difference between groups was mitigated by including age in the statistical analyses. Additionally, we performed statistical analyses on a subset of subjects, limiting the age range in both groups to 18–40 years old, leaving 14 non-SCD and 12 SCD subjects. By eliminating the age difference between groups, the significant differences in BM_v_ and BM_n-v_ between SCD and non-SCD were preserved. We further examined, in the same subset of subjects, the effect of baseline blood pressure by including it as another predictor in the multiple linear regression. Again, the significant differences in BM_v_ and BM_n-v_ between SCD and non-SCD were preserved.

The last point that should be addressed relates to the reason for dividing the subjects into non-sickle disease and sickle cell disease. The healthy subjects (AA) and sickle cell trait (heterozygous hemoglobin AS) subjects were combined to form the non-SCD group because AS patients are identical to AA subjects in all ways except they have HbS. They do not sickle at all under the circumstances of this study. The SCD group consisted of non-transfused subjects and subjects receiving chronic transfusion therapy. These patients have sickling ongoing, albeit at different levels. In a separate analysis, we stratified these subjects into 4 different groups: AA, AS, non-transfused SCD and chronically transfused SCD subjects. We could not detect any statistical difference between AA and AS subjects in any of the hematological parameters or the biophysical markers under investigation. Similarly, for the SCD groups we also could not detect the effects on chronic transfusion on the pain response.

## Conclusions

In summary, we employed a mathematical model, from which we derived biophysical markers, to determine the relative importance of the key functional mechanisms involved in pain-induced vasoconstriction in SCD subjects and non-SCD controls. The fact that we found little group difference in the neurogenic component, represented by marker BM_n_, was rather unexpected. On the other hand, it was the blood pressure-related markers, BM_v_ and BM_n-v_, that showed strong differences between SCD and non-SCD. These biophysical markers provide a means of phenotyping severity of disease in sickle-cell anemia that is based more on underlying physiology than on genotype. Our approach is particularly useful from a clinical standpoint as it is based on measurements that are necessarily noninvasive and acquired in the form of continuously recorded time-series.

## Supporting information

S1 Fig **Representative data recorded during a test procedure in (a) a non-SCD subject and (b) a SCD subject showing similarity between changes finger blood volume and microvascular blood flow.** The top row shows changes in temperature (ΔTemp, °C). 0°C indicates no heat was delivered. Row 2 shows changes in beat-averaged blood pressure (MAP, mmHg). Row 3 shows changes in tidal volume (ΔV_T_, L). Row 4 shows corresponding changes in finger blood volume (ΔFBV, %). The bottom row shows microvascular blood flow measured by laser Doppler flowmetry (ΔPU, perfusion unit).
(TIF)

S1 Table Subject characteristics, baseline physiological measurements and biophysical markers of the study cohort. (XLSX)

S1 File Details on modeling FBV response to heat-induced pain and model estimation. (DOCX)
