# Supplementary figures and images for: Biophysical markers of the peripheral vasoconstriction response to pain in sickle cell disease
Source: PLoS One. 2017 May 24;12(5):e0178353. doi: 10.1371/journal.pone.0178353 (PMC5443571; doi:10.1371/journal.pone.0178353)

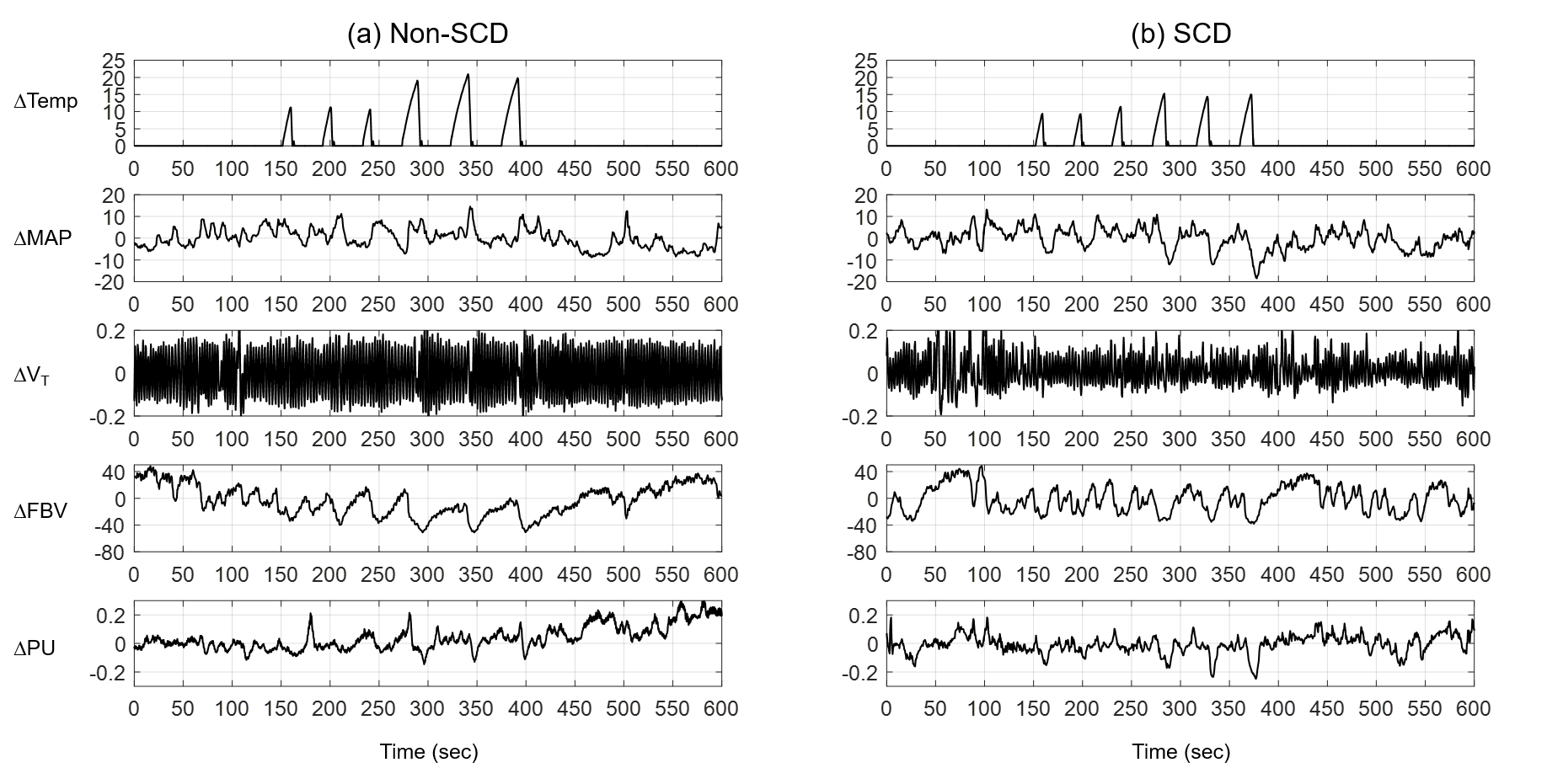

Supplement: S1 Fig — Representative data recorded during a test procedure in (a) a non-SCD subject and (b) a SCD subject showing similarity between changes finger blood volume and microvascular blood flow. The top row shows changes in temperature (ΔTemp, °C). 0°C indicates no heat was delivered. Row 2 shows changes in beat-averaged blood pressure (MAP, mmHg). Row 3 shows changes in tidal volume (ΔVT, L). Row 4 shows corresponding changes in finger blood volume (ΔFBV, %). The bottom row shows microvascular blood flow measured by laser Doppler flowmetry (ΔPU, perfusion unit). (TIF) [file pone.0178353.s001.tif]
