# Supplementary material for: Biophysical markers of the peripheral vasoconstriction response to pain in sickle cell disease
Source: PLoS One. 2017 May 24;12(5):e0178353. doi: 10.1371/journal.pone.0178353 (PMC5443571; doi:10.1371/journal.pone.0178353)
Supplement: S1 File — (DOCX) [file pone.0178353.s003.docx]

# Supporting Information

## Modeling FBV response to heat-induced pain

To unravel the major contributing mechanisms to pain-induced vasoconstriction, we proposed 3 model configurations based on our understanding of the physiology regulating FBV.

### Model A

Model A assumes that the regulation of ΔFBV is governed by two major functional mechanisms: blood pressure coupling (BPC) and respiratory coupling (RPC), which relates ΔMAP and ΔV_T_ to ΔFBV, respectively. Further, the model assumes that heat-induced pain modulates ΔMAP and ΔV_T_ such that the fluctuations of MAP and V_T_ reflect the effect of pain, which then affected ΔFBV. The mathematical representation is as follows

|  | $\Delta FBV\left( t \right)=\sum_{i=0}^{M-1} h_{RPC}\left( i \right)\Delta V_{T}\left( t-i-T_{RPC} \right)$ $+\sum_{i=0}^{M-1} h_{BPC}\left( i \right)\Delta MAP\left( t-i-T_{BPC} \right)$ $+\varepsilon_{FBV}\left( t \right) .$ | (1) |
| --- | --- | --- |

### Model B

Model B assumes that heat-induced pain affects ΔFBV through sympathetic modulation directly as well as through its modulation on MAP and V_T_. Thus, ΔTemp (representing changes in pain intensity) was incorporated as the third input as neurogenic thermal pain coupling (THM). The mathematical representation is as follows

|  | $\Delta FBV\left( t \right)=\sum_{i=0}^{M-1} h_{RPC}\left( i \right)\Delta V_{T}\left( t-i-T_{RPC} \right)$ $+\sum_{i=0}^{M-1} h_{BPC}\left( i \right)\Delta MAP\left( t-i-T_{BPC} \right)$ $+\sum_{i=0}^{M_{THM}-1} h_{THM}\left( i \right)\Delta Temp\left( t-i-T_{THM} \right)$ $+ \varepsilon_{FBV}\left( t \right) .$ | (2) |
| --- | --- | --- |

### Model C

Model C incorporates the interaction effect between ΔMAP and ΔTemp on ΔFBV, reflecting the neurogenic-vascular interaction, and is described in the Methods section.

| $\Delta FBV\left( t \right)=\sum_{i=0}^{M-1} h_{RPC}\left( i \right)\Delta V_{T}\left( t-i-T_{RPC} \right)$ $+\sum_{i=0}^{M-1} h_{BPC}\left( i \right)\Delta MAP\left( t-i-T_{BPC} \right)$ $+\sum_{i=0}^{M_{THM}-1} h_{THM}\left( i \right)\Delta Temp\left( t-i-T_{THM} \right)$ $+\sum_{i=0}^{M-1} \sum_{j=0}^{M_{THM}-1} h_{BPCTHM}\left( i,j \right)\Delta MAP\left( t-i-T_{BPC} \right)\Delta Temp\left( t-j-T_{THM} \right)$ $+ \varepsilon_{FBV}\left( t \right) .$ | (3) |
| --- | --- |

We assumed that the FBV response to heat-induced pain could be approximated by a linear and time-invariant model such that each input-output functional mechanism in the proposed models could be represented by an impulse response. An impulse response, *h*, quantifies the dynamics in the output as a result of a brief and abrupt unit increase in an input. For example, an impulse response relating ΔTemp to ΔFBV, *h_THM_*, represents FBV dynamics when there is a brief and abrupt increase in Temp by 1 °C. In effect, an impulse response provides a “standardized” dynamic response to a unit input, allowing fair comparison across subjects. When there are multiple functional mechanisms in a model, each impulse response represents the standardized dynamic response to the unit input after accounting for other input effects. For example, *h_THM_* in Model B represents FBV dynamics resulting from a unit increase in temperature after accounting for effects of blood pressure and respiration on FBV. For other parameters in equation (1)-(3), *ε_FBV_* denotes extraneous influences on ΔFBV that could not be explained by the model; *T* denotes the pure delay or the time it takes for the input to affect an output; and *M* and *M_THM_* denote system memory or the maximum duration it takes for the changes in the output caused by an abrupt increase in the input to persist. Based on the lengths of our datasets and preliminary analyses, we found the system memory of 30 seconds to be a suitable choice for the RPC and BPC mechanisms, and 100 seconds for THM. The model components, i.e. the impulse responses, were estimated from the collected data: 5 minutes of baseline to 4 minutes after the end of the last pain pulse. Section 2 details of model estimation procedures.

### Model selection

For each model candidate, we calculated the predicted FBV response and the goodness of fit (R^2^) to see which model configuration could best capture the variability in ΔFBV. To account for the model complexity, we employed F-test to check if one model configuration fit data statistically better than another [1]. Model A was able to explain 25.3 ± 2.5% (mean ± SEM) of variability in FBV (i.e. R^2^ = 0.25 ± 0.025). After incorporating the direct effect of heat-induced pain, Model B was able to explain 42.9 ± 2.3% of FBV variability, an improvement by almost two folds from Model A. Next, with the additional interaction effect between ΔMAP and ΔTemp in Model C, the model was able to capture 49.0 ± 2.3% variability in FBV. Based on the R^2^, these results suggest that BPC (relating ΔMAP to ΔFBV) and THM (relating ΔTemp to ΔFBV) components were the major contributing mechanisms to vasoconstriction response to heat pain. Although the improvement in the model fit by incorporating the interaction effect seemed relatively small compared to the contribution by the two aforementioned mechanisms, F-test showed that Model C had significantly better fit than model B in all subjects. Therefore, Model C was selected as the best model that could capture the vasoconstriction response due to heat-induced pain and all subsequent analyses were based on this model. Note that we also tested whether R^2^ was different between groups and found no significant difference. As such, the R^2^ results reported in this section combined both non-SCD and SCD subjects together.

## Model estimation

Assuming that the system under investigation was linear and time-invariant, each functional mechanism could be represented by an impulse response. In linear systems theory, the impulse response provides a complete characterization of the dynamic properties of the system because the response of this system to any arbitrary input can be predicted by convolving the input with the impulse response [2]. In this study, we employed the basis function expansion technique to estimate an impulse response – that is each impulse response can be represented as a weighted sum of a set of orthonormal basis functions. The advantage of this technique is that it greatly reduces the number of parameters to be estimated and thus improves estimation accuracy. With the improved estimation robustness, this technique can be applied on relatively short and noise-contaminated data where the inputs do not have to be strictly white and Gaussian [3]. We chose Meixner function, or also known as generalized Laguerre function, as the basis function for the impulse responses [4,5]. Meixner function has exponential decaying form, making it suitable for modeling an impulse response as an impulse response of a stable system always decays to zero. Another parameter of the Meixner function called order of generalization allows us to have control over the rise time of the function, making it suitable for modeling impulse responses with slow initial dynamics. The higher the order of generalization, the slower the rise time. The equation of the impulse response is as follows

|  | $h_{x}\left( t \right)= \sum_{i=1}^{q_{x}} c_{x}\left( i \right)B_{i}^{\left( n_{x} \right)}\left( t \right).$ | (4) |
| --- | --- | --- |

The interaction kernel can be written as

|  | $h_{x_{1}x_{2}}\left( t_{1},t_{2} \right)= \sum_{i=1}^{q_{x_{1}}} \sum_{j=1}^{q_{x_{2}}} c_{x_{1}x_{2}}\left( i,j \right)B_{i}^{\left( n_{x_{1}} \right)}\left( t_{1} \right)B_{j}^{\left( n_{x_{2}} \right)}\left( t_{2} \right) .$ | (5) |
| --- | --- | --- |

*h_x_* represents the impulse response relating input *x* to the output. In this case, *x* represents ΔV_T_, ΔMAP, or ΔTemp. *h_x_*_1_*_x_*_2_ represents the dynamic interaction between input *x_1_* and *x_2_* on the output, where *x_1_* represents ΔMAP and *x_2_* represents ΔTemp. *q_x_*, *q_x_*_1_ and *q_x_*_2_ are the number of basis functions used in the expansion of the impulse responses and interaction kernel. *B_i_*^(^*^n^*^)^(*t*) and *B_j_*^(^*^n^*^)^(*t*) are the orthonormal sets of Meixner functions with *n*^th^ order of generalization. *c_x_* represents the expansion coefficients of the basis functions for the impulse response and and *c_x_*_1_*_x_*_2_ represents the expansion coefficients of the interaction kernel.

Substitute *h_x_* into equation (1)-(3) and *h_x_*_1_*_x_*_2_ into equation (3), we could rewrite Model A, B and C as follows:

Model A:

|  | $\Delta FBV\left( t \right)=\sum_{i=1}^{q_{RPC}} c_{RPC}\left( i \right)u_{i}(t) +\sum_{i=1}^{q_{BPC}} c_{BPC}(i)v_{i}(t) +\varepsilon_{FBV}\left( t \right)$ | (6) |
| --- | --- | --- |

Model B:

|  | $\Delta FBV\left( t \right)=\sum_{i=1}^{q_{RPC}} c_{RPC}\left( i \right)u_{i}\left( t \right) +\sum_{i=1}^{q_{BPC}} c_{BPC}\left( i \right)v_{i}\left( t \right)$ $+\sum_{i=1}^{q_{THM}} c_{THM}\left( i \right)w_{i}\left( t \right) +\varepsilon_{FBV}\left( t \right)$ | (7) |
| --- | --- | --- |

Model C:

|  | $\Delta FBV\left( t \right)=\sum_{i=1}^{q_{RPC}} c_{RPC}\left( i \right)u_{i}\left( t \right) +\sum_{i=1}^{q_{BPC}} c_{BPC}\left( i \right)v_{i}\left( t \right) +\sum_{i=1}^{q_{THM}} c_{THM}\left( i \right)w_{i}\left( t \right)$ $+\sum_{i=1}^{q_{BPC}} \sum_{j=1}^{q_{THM}} c_{BPCTHM}(i,j)v_{i}\left( t \right)w_{j}\left( t \right)+\varepsilon_{FBV}\left( t \right)$ | (8) |
| --- | --- | --- |

where *u_i_*(*t*), *v_i_*(*t*) and *w_i_*(*t*) (or *w_j_*(*t*)) are the convolution of the basis functions with ΔV_T_, ΔMAP and ΔTemp, respectively. The convolution between the basis functions and the inputs are as follows:

|  | $u_{i}\left( t \right)=\sum_{\tau=0}^{M-1} B_{i}^{\left( n_{RPC} \right)}\left( i \right)\Delta V_{T}\left( t-\tau-T_{RPC} \right)$ | (9) |
| --- | --- | --- |
|  | $v_{i}\left( t \right)=\sum_{\tau=0}^{M-1} B_{i}^{\left( n_{BPC} \right)}\left( i \right)\Delta MAP\left( t-\tau-T_{BPC} \right)$ | (10) |
|  | $w_{i}\left( t \right)=\sum_{\tau=0}^{M_{THM}-1} B_{i}^{\left( n_{THM} \right)}\left( i \right)\Delta Temp\left( t-\tau-T_{THM} \right) .$ | (11) |

The unknown coefficients *c_RPC_*, *c_BPC_*, *c_THM_* and *c_BPCTHM_* in equation (6)-(8) were estimated using least-squares minimization. The estimation was repeated for all possible combinations of model parameters *T*, *q* and *n* (Table 1 in S1 File). In order to determine the optimal parameter set, minimum description length (MDL) was computed for all parameter combinations.

|  | $MDL=\log\left( \frac{J_{R}}{J_{y}} \right)+q_{tot} \times\frac{log(L)}{L}$ | (12) |
| --- | --- | --- |

*J_R_* is the variance of the residual errors between the observed and predicted output and *J_y_* is the variance of the observed output. *q_tot_* is the total number of unknown coefficients to be estimated. For Model A, *q_tot_* = *q_RPC_* + *q_BPC_*; for Model B, *q_tot_* = *q_RPC_* + *q_BPC_* + *q_THM_*; and for Model C, *q_tot_* = *q_RPC_* + *q_BPC_* + *q_THM_* + (*q_BPC_* × *q_THM_*). Lastly, *L* is the number of data samples in the output.

MDL gives a measure of balance between how well the model fits the data and the model complexity [6]. It decreases as the model fit improves but increases as the number of parameters in the model increases (increasing model complexity). The optimal set of parameters was selected based on the global search for minimal MDL. Using the optimal parameter set, the expansion coefficients were computed and the impulse responses and interaction kernel were then estimated using equation (4) and (5).

**Table 1. Model parameters**

|  | ***T* (seconds)** | ***q*** | ***n*** |
| --- | --- | --- | --- |
| RPC | 0 – 2 | 3 | 1 – 4 |
| BPC | 0 – 5 | 4 | 0 – 6 |
| THM | 0 – 5 | 4 | 1 – 6 |

*Definition of abbreviations:* T = pure delay; q = number of basis functions; n = order of generalization; RPC = respiratory coupling; BPC = blood pressure coupling; THM = thermal pain coupling.

## References

1. Hamilton JD. Time Series Analysis. 1st ed. Vol. 39, Book. Princeton, New Jersey: Princeton University Press; 1994. 820 p.

2. Khoo MCK. Physiological Control Systems: Analysis, Simulation and Estimation. Piscataway, NJ: Wiley/IEEE Press; 2000.

3. Marmarelis VZ. Identification of nonlinear biological systems using Laguerre expansions of kernels. Ann Biomed Eng. 1993/11/01. 1993;21(6):573–89.

4. Brinker AC Den. Meixner-like functions having a rational z-transform. Int J Circuit Theory Appl. 1995 May;23(3):237–46.

5. Asyali MH, Juusola M. Use of meixner functions in estimation of Volterra kernels of nonlinear systems with delay. IEEE Trans Biomed Eng. 2005/02/16. 2005;52(2):229–37.

6. Rissanen J. Modeling by shortest data description. Automatica. 1978;14:465–71.
